# Relationship between continuous infusion meropenem PK/PD target attainment and C‐reactive protein dynamics in onco‐haematologic patients with febrile neutropenia

**DOI:** 10.1002/bcp.70667

**Published:** 2026-07-02

**Authors:** Chun Liu, Pier Giorgio Cojutti, J. G. Coen van Hasselt, Michele Bartoletti, Maddalena Giannella, Pierluigi Zinzani, Pierluigi Viale, Federico Pea

**Affiliations:** ^1^ Department of Medical and Surgical Sciences Alma Mater Studiorum, University of Bologna Bologna Italy; ^2^ Clinical Pharmacology Unit IRCCS Azienda Ospedaliero‐Universitaria di Bologna Bologna Italy; ^3^ Division of Systems Biomedicine and Pharmacology, Leiden Academic Centre for Drug Research Leiden University Leiden The Netherlands; ^4^ Infectious Diseases Unit IRCSS Humanitas Research Hospital Rozzano Italy; ^5^ Department of Biomedical Sciences Humanitas University Pieve Emanuele Italy; ^6^ Infectious Diseases Unit, Department for Integrated Infectious Risk Management IRCCS Azienda Ospedaliero‐Universitaria di Bologna Bologna Italy; ^7^ Istituto di Ematologia “Seràgnoli” IRCCS Azienda Ospedaliero‐Universitaria di Bologna Bologna Italy

**Keywords:** C‐RP, meropenem, population PK/PD

## Abstract

**Aim:**

Meropenem is a second‐line antimicrobial agent in the treatment of febrile neutropenia in onco‐haematological patients. A population pharmacokinetic/pharmacodynamic (PK/PD) analysis for assessing the relationship between meropenem exposure and C‐reactive protein (C‐RP) over time was conducted.

**Methods:**

Non‐linear mixed‐effect modelling was used to fit meropenem and C‐RP concentrations. Monte Carlo simulations were conducted to assess median percentage reduction of C‐RP from baseline associated with continuous infusion meropenem dosing, ensuring optimal PK/PD target attainment against *Pseudomonas aeruginosa* and *Enterobacterales*, namely, 100%*t*_>4 × MIC_. Clinical outcome was assessed at the end of antimicrobial treatment.

**Results:**

A total of 141 patients were included in the analysis. A one‐compartment model with estimated glomerular filtration rate as a covariate on drug clearance well‐described meropenem pharmacokinetics. An indirect turnover maximum inhibition model was used to capture C‐RP dynamics. Patients with C‐RP Day 4/baseline ratio of <0.4 (fast response), between 0.4 and 0.8 (slow response) and >0.8 (no response) were 8.5%, 15.6% and 75.9%, respectively. Regression analysis showed that patients with fast and slow response patterns were associated with favourable clinical outcomes. Simulations showed that rapid and slow pattern patients reached C‐RP reduction >90% from baseline at Days 6 and 10, respectively. No response pattern had C‐RP peaking at Day 3 and eventually gradually reducing within 2 weeks.

**Conclusion:**

Despite optimal meropenem PK/PD target attainment, C‐RP decreases in no more than one‐fourth of onco‐haematological patients during the first 2 weeks of treatment. Among no‐response patients, C‐RP should be associated with other biomarkers to discriminate subjects with favourable clinical outcome.

**What is already known about this subject**

- Meropenem is used as a second‐line antipseudomonal agent in onco‐haematological patients with febrile neutropenia.
- Assessing clinical response to antimicrobial therapy in this population is based on clinical evaluation and assessment of inflammatory biomarkers of infection.
- C‐RP dynamics may be influenced by several pathophysiological conditions in patients with febrile neutropenia.

**What this study adds**

- C‐RP reduction by Day 4 may predict response to meropenem therapy.
- However, only 1/4 of onco‐haematological patients had a C‐RP ratio <0.4 (fast response) and between 0.4 and 0.8 (slow response), despite optimal meropenem exposure.
- Among patients with no C‐RP response, other biomarkers should be investigated to discriminate subjects with favourable clinical outcome.

## 1 INTRODUCTION

Onco‐haematological patients undergoing induction chemotherapy or conditioning regimens for stem cell transplantation are vulnerable to febrile neutropenia. Febrile neutropenia is related to bloodstream infection caused by Gram‐negatives in as much as 11%–38% of cases.^1^ Antimicrobial agents conventionally used in onco‐haematological patients should target both *Pseudomonas aeruginosa* and *Enterobacterales*, such as *Escherichia coli* and *Klebsiella pneumoniae*.^2^

International guidelines recommend an antipseudomonal beta‐lactam, namely, piperacillin‐tazobactam, ceftazidime or cefepime as first‐line treatment of febrile neutropenia in onco‐haematological patients.^3^, ^4^ Escalation to meropenem is recommended whenever no clinical response occurs within 48–72 h from starting treatment.

Meropenem is a carbapenem whose pharmacodynamic activity is maximized when plasma concentrations are maintained 4‐fold higher than the pathogen minimum inhibitory concentration (MIC) for 100% of the dosing interval (100%*t*_>4 × MIC_), defined as an aggressive PK/PD target.^5^, ^6^ Administration by continuous infusion (CI) coupled with a therapeutic drug monitoring (TDM)–guided approach may be highly beneficial for attaining an aggressive PK/PD target with meropenem.^7^, ^8^

Because febrile neutropenia need empirical treatment, plasma concentration should be targeted at least at 8 mg/L for maximizing the likelihood of aggressive PK/PD target attainment against all of the susceptible pathogens, namely, a 100%*t*_>4 × MIC_ against pathogens with an MIC value up to the EUCAST clinical breakpoint of 2 mg/L.^8^, ^9^ Assessing clinical response to antimicrobial treatment is quite challenging in this population due to the lack of reliable biomarkers of response. Evaluation of clinical response may be based on monitoring either signs and symptoms of infection or inflammatory biomarkers, namely, C‐reactive protein (C‐RP).^10^, ^11^ C‐RP is an acute‐phase protein extensively used as a biomarker of evolution of bacterial infections.^12^, ^13^ Unfortunately, the role of C‐RP in managing febrile neutropenia may be biased by some pathophysiological conditions such as the engraftment status and the integrity of the immune system. To the best of our knowledge, no study has assessed up to date the relationship between C‐RP dynamics over time and aggressive PK/PD target attainment of meropenem among onco‐haematological patients with febrile neutropenia.

The aim of this study was to develop a population PK/PD model to describe the relationship between C‐RP dynamics and aggressive PK/PD target attainment of meropenem over time in a cohort of onco‐haematological patients having febrile neutropenia.

## 2 METHODS

### 2.1 Study design

This was a retrospective, single‐centre, study that was carried out among adult onco‐haematological patients admitted at the Division of Hematology of the IRCCS Azienda Ospedaliero‐Universitaria di Bologna, Italy, between January 2021 and July 2023 and receiving TDM‐guided therapy of CI meropenem for treating febrile neutropenia. The study was approved by the Ethics Committee of the Azienda Ospedaliero‐Universitaria di Bologna (No. 308/2021/Oss/AOUBo). Written informed consent was waived due to the retrospective nature of this investigation.

Meropenem therapy was started with a loading dose of 2 g over 30 min followed by a maintenance dose of 1 g q8h over 8 h or 0.5 g q6h over 6 h (viz., by CI) in patients having estimated glomerular filtration rate (eGFR) of ≥60 or <60 mL/min/1.73 m^2^, respectively, with an expected treatment duration of at least 4 days. Estimated glomerular filtration rate was calculated by means of the Chronic Kidney Disease Epidemiology (CKD‐EPI) formula.^14^ Stability of CI meropenem in aqueous solution was granted by reconstituting each solution every 6–8 h and infusing over 6–8 h.^15^ At steady‐state, all patients underwent a real‐time therapeutic drug monitoring (TDM)–based program for adjusting exposure finalized at achieving meropenem steady‐state concentration (Css) of 8–16 mg/L, namely, a Css/MIC ratio of 4‐ to 8‐fold the EUCAST clinical breakpoint of meropenem against the *Enterobacterales* and *P. aeruginosa*, namely, 2 mg/L.

TDM was assessed first after at least 48 h from starting therapy and reassessed every 48–72 h. At each TDM assessment, 3 mL of peripheral blood was collected, and meropenem concentrations were measured by means of a validated liquid chromatography–tandem mass spectrometry (LC–MS/MS).^16^ The lower limit of quantification (LLOQ) was 0.3 mg/L. The LLOQ of C‐RP was 0.1 mg/dL. The following demographic and clinical data were retrieved from each patient's medical record: age, gender, weight, height, type of haematologic disease and coadministered antimicrobials. Serum creatinine, meropenem dose and C‐RP concentrations were collected at each TDM assessment and up to the end of meropenem treatment.

C‐RP individual patterns were classified according to the definition proposed by Povoa et al. in the critically ill setting,^17^ in which four individual patterns of response to antibiotic therapy based on C‐RP changes from baseline to Day 4 (defined as C‐RP ratio) were described: fast response pattern (C‐RP ratio to <0.4), slow response pattern (C‐RP ratio between 0.4–0.8), non‐response pattern (C‐RP ratio >0.8) and biphasic response pattern (initial drop to C‐RP ratio <0.8 followed by a re‐rise above this threshold).

### 2.2 Population pharmacokinetic/pharmacodynamic analysis

An intermediate PK/PD approach was used in model building for overcoming the initial model instability related to the simultaneous fitting of both the PK and the PD data. At first, the PK model was built, and subsequently, the PD model was developed and fitted to the individual C‐RP concentrations over time. In this PD model, the PK parameters were fixed to the population values and to the standard deviations of the random effects that were estimated in the PK model. Both the PK and the PD population models were developed by means of the nonlinear mixed‐effect model implemented in the Monolix software (Version 2023R; Lixoft, Antony, France).

Since all meropenem concentrations have been collected during CI administration, a one‐compartment pharmacokinetic model including eGFR as a covariate on total meropenem clearance was used, according to previous works.^8^, ^18^ Volume of distribution was fixed to 28.5 L, based on Contejean et al.^18^ The structural PK/PD model was an indirect turnover model with inhibition of C‐RP production, as follows:

$$\mathrm{dR}/\mathrm{dt}=k_{\text{in}}*\left(1-\frac{\mathrm{Cc}}{\mathrm{Cc}+\mathrm{IC}50}\right)*e^{-k*t}-k_{\text{out}}*R,$$

where *R* represents the response (i.e., C‐RP concentration in plasma); *dR/dt* represents the changing rate of C‐RP in plasma relative to time; *Cc* is meropenem plasma concentration; *k*_in_ and *k*_out_ represent the C‐RP production and degradation rate constants, respectively; *IC*_50_ represents the meropenem concentration causing half‐maximal rate of C‐RP reduction; and *k* is a constant introduced to discriminate between patients with an upward or downward trend of C‐RP over time.

Exponential random effects were assumed to describe between‐subject variability. Correlations between random effects were tested in the variance–covariance matrix and implemented into the structural model accordingly. Constant, proportional or combined error models were tested for describing the residual variability. A log‐normal distribution of population parameters was applied. Covariate analysis was conducted according to a forward/backward process. In the forward step, the correlation between each covariate and the random effect of the estimated PK parameter was tested by means of Pearson's correlation test. In the backward step, each covariate was tested for potential removal from the full covariate model based on the results of the Wald test. A covariate was included in the final model if a decrease of ≥3.84 points in the objective function value (OFV), coupled with a reduction in the Akaike information criterion (AIC), was observed with respect to the base model. Type of onco‐haematological disease, concomitant treatment with antifungals, concomitant treatment with anti‐Gram‐positive agents, treatment duration, weight, BMI and gender were tested as potential covariates in the PK/PD model.

### 2.3 Model evaluation

Reliability of the PK/PD model was based on having relative standard errors (RSE%) of the PK and PD estimates <30%–40% and on the adequacy of the standard goodness‐of‐fit plots, namely, observation‐versus‐population and observation‐versus‐individual predictions, usual residual‐based plots (individual‐ and population‐weighted residuals) and prediction‐corrected visual predictive check (pc‐VPC). The pc‐VPC shows the time course of the 10th, the 50th and the 90th percentiles of the observations and the corresponding 90% prediction intervals calculated from 10 000‐subject Monte Carlo simulation samples. Generally, PK/PD models are considered reliable if the 10th, the 50th and the 90th percentiles of observed data are inside the simulated prediction intervals. Moreover, 1000 non‐parametric bootstrap iterations with resampling for each estimated PK and PD parameter were simulated using the Rsmlx package of R (R speaks Monolix), and the median (interquartile range) was reported. The normalized prediction distribution error (NPDE) and the numerical predictive check (NPC) graph were also evaluated.

### 2.4 Monte Carlo simulations

One‐thousand‐subject Monte Carlo simulations based on the final PK/PD model were performed in Simulx (Version 2023R1; Lixoft, Antony, France) in order to assess the median of the percentage reduction or increase of C‐RP at each day versus baseline according to three patterns of C‐RP, namely, (i) the fast response pattern, (ii) the slow response pattern and (iii) both the non‐response and the biphasic response pattern.

The meropenem dosages used in simulations were those that ensured a Css/MIC > 4 in febrile neutropenic patients with haematologic malignancies,^8^ namely, 1 g every 8 h for eGFR class of 50–89 mL/min/1.73 m^2^, 1 g every 6 h for eGFR of 90–129 mL/min/1.73 m^2^ and 1.5 g every 6 h for eGFR of ≥130 mL/min/1.73 m^2^.

### 2.5 Clinical outcome

Clinical outcome at end of therapy was assessed by two infectious diseases consultants based on treatment response and according to the criteria reported by.^19^ Clinical outcome was defined as cured, improved, unchanged or failed. Cured was defined as the composite achievement of all of the following criteria: defervescence lasting ≥24 h, microbiological eradication documented by negative blood cultures (only in case of microbiologically documented infections), resolution of the signs and symptoms of infection and no need for escalation therapy. Improved was defined as the achievement of at least two out of the aforementioned criteria. Unchanged or failed were defined as the absence of achievement of any of the aforementioned criteria or as worsening of the clinical conditions, respectively. Patients having clinical cure or improvement were grouped together and considered as having a favourable clinical outcome. Conversely, those unchanged or failed were grouped together and considered as having a negative clinical outcome.

### 2.6 Statistical analysis

The Kolmogorov–Smirnov test was used to assess whether data were normally or non‐normally distributed. Accordingly, the mean ± SD or median with IQR were used in the descriptive statistics. Categorical variables were compared by the ×2 test with Yates's correction or Fisher's exact test as necessary, and continuous variables were compared using the Student's t‐test or Mann–Whitney test. A *p*‐value of >.05 was required to achieve statistical significance. Univariate and multivariate linear regression analyses were performed to identify independent predictors of favourable clinical outcome. Multivariate stepwise backward analysis included all variables significant at *p* ≤ .2 in the univariate analysis. All statistical analyses were performed with R version 4.3.3.

## 3 RESULTS

### 3.1 Demographics and clinical data

A total of 174 patients were initially screened (Figure 1), and 141 out of them were deemed eligible for the PK/PD analysis (59.6% males, 84/141). The vast majority of the population was co‐treated with antifungals (95.0%, 134/141). Eighty‐one patients (57.4%, 81/141) received also an anti‐Gram‐positive agent. Patient characteristics are summarized in Table 1. Median (min–max range) age, weight and eGFR were 59 (19–81) years, 70 (42–118) kg and 97.0 (17.0–195.6) mL/min/1.73 m^2^, respectively. Acute myeloid leukaemia was the most prevalent underlying haematological disease (64.5%, 91/141). Microbiological isolates were identified in 32.6% of the population (46/141). Median (min–max) duration of meropenem treatment was 10 (2–32) days. Median (min–max) number of meropenem and C‐RP measurements per patient were 2 (1–7) and 12 (1–32), respectively. Median (min–max) meropenem Css and C‐RP concentration at baseline were 11.4 (3.0–39.5) mg/L and 9.8 (4.9–17.4) mg/dL, respectively.

**FIGURE 1:**
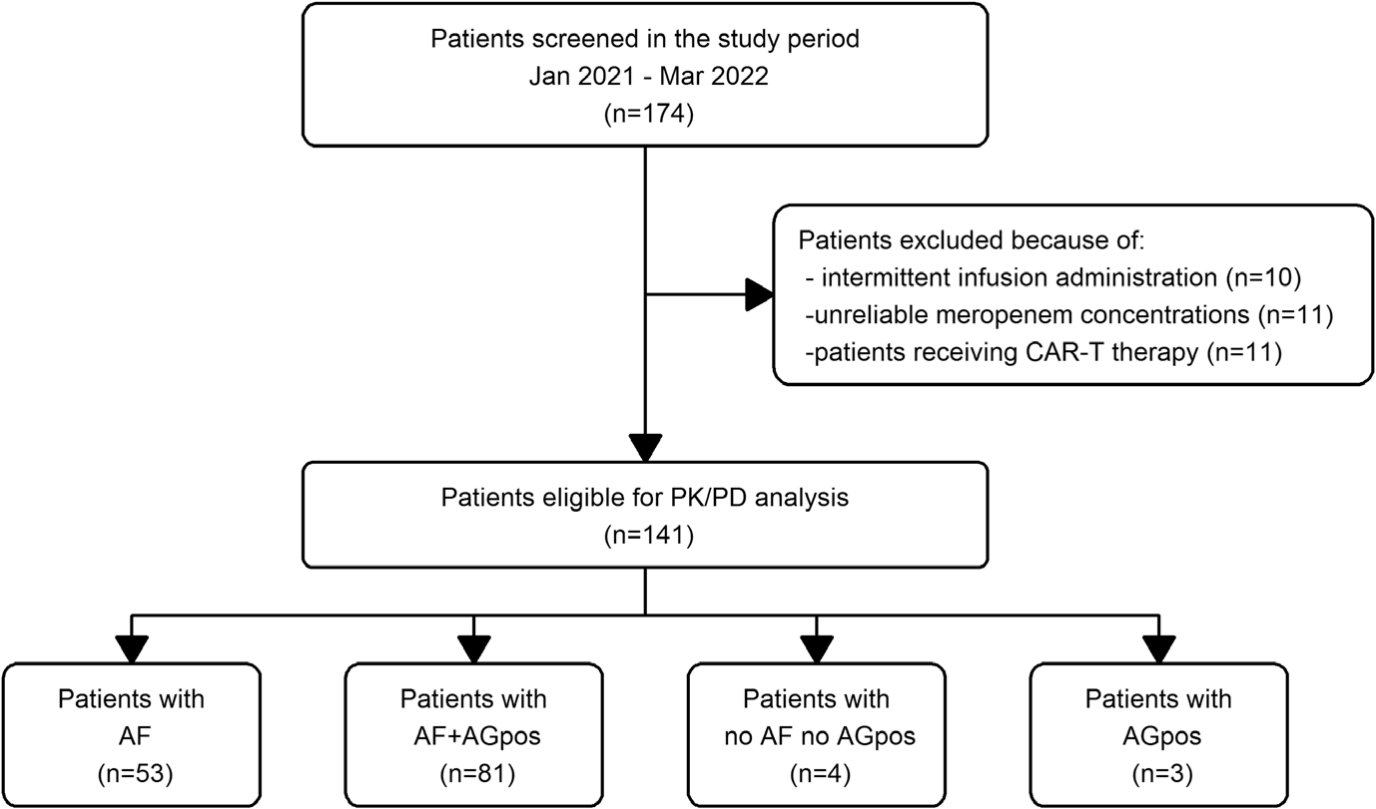
Flowchart of patients' inclusion criteria in the PK/PD study. AF, antifungal agent; AGpos, anti‐Gram‐positive agent.

**TABLE 1:** Demographics and clinical characteristics of the population.

Number of subjects	141
Gender (female/male)	57/84 (40.4/59.6)
Age (years)	59 (52–64)
Weight (kg)	70 (62–80)
Height (m)	1.70 (1.65–1.79)
eGFR (mL/min/1.73 m^2^)	97.0 (80.0–109.0)
Underlying disease	
AML	91 (64.5)
NHL	23 (16.3)
ALL	9 (6.4)
MM	6 (4.3)
CML	3 (2.1)
CLL	2 (1.4)
Other	7 (4.9)
Meropenem treatment	
Meropenem total dose	1 g q6h (1 g q8h to 1 g q6h)
Treatment duration (days)	10 (7–13)
Meropenem Css (mg/L)	11.4 (8.05–16.45)
Pharmacodynamics	
C‐RP baseline value, mg/dL	9.8 (4.9–17.4)
No of subsequent C‐RP assessments per patient	12 (1–32)

*Note*: Data are expressed as median (IQR) for continuous variables and number (%) for dichotomous variables.

Abbreviations: ALL, acute lymphoid leukaemia; AML, acute myeloid leukaemia; CLL, chronic lymphoid leukaemia; CML, chronic myeloid leukaemia; Css, meropenem steady‐state concentration; eGFR, estimated glomerular filtration rate; MM, multiple myeloma; NHL, non‐Hodgkin lymphoma.

Clinical outcome was available for 49.6% (70/141) patients. Of them, 74.3% (52/70) had a favourable clinical outcome (26.9% [14/52] and 73.1% [38/52] were cured and improved, respectively), whereas 25.7% (18/70) had either no clinical response or treatment failure.

### 3.2 Population PK/PD modelling

A total of 283 meropenem plasma Css and 1373 plasma C‐RP concentrations were included in the PK/PD model. A one‐compartment model with zero‐order administration and first‐order elimination from the central compartment was developed, and eGFR was included as a covariate on meropenem CL in the final population PK model. The goodness of fit of the observed concentrations versus both the population‐predicted and the individual‐predicted concentrations of meropenem and C‐RP were reported in Figure 2 (upper and bottom panels, respectively). The pc‐VPCs showed good predictive performance for both the PK (Figure 3A) and the PD model (Figure 3B), as witnessed by the fact that most concentrations fell within the prediction intervals. The distributions of the individual weighted residuals versus individual predictions of both meropenem and C‐RP concentrations were homogeneous, showing no specific trend (Figures S1 and S2). The NPDE graphs showed a normal distribution for both the PK and the PD components (Figures S3 and S4). For most of the meropenem and C‐RP concentrations, the NPC graphs showed that the theoretical cumulative distribution of the observations was close to the empirical distribution (Figures S5 and S6), with a slight underprediction at the lower values. None of the concentrations detected in our population for either meropenem or C‐RP was below the respective LLOQ (min–max range 3–39.5 mg/L for meropenem; 0.15–65.9 mg/dL for C‐RP).

**FIGURE 2:**
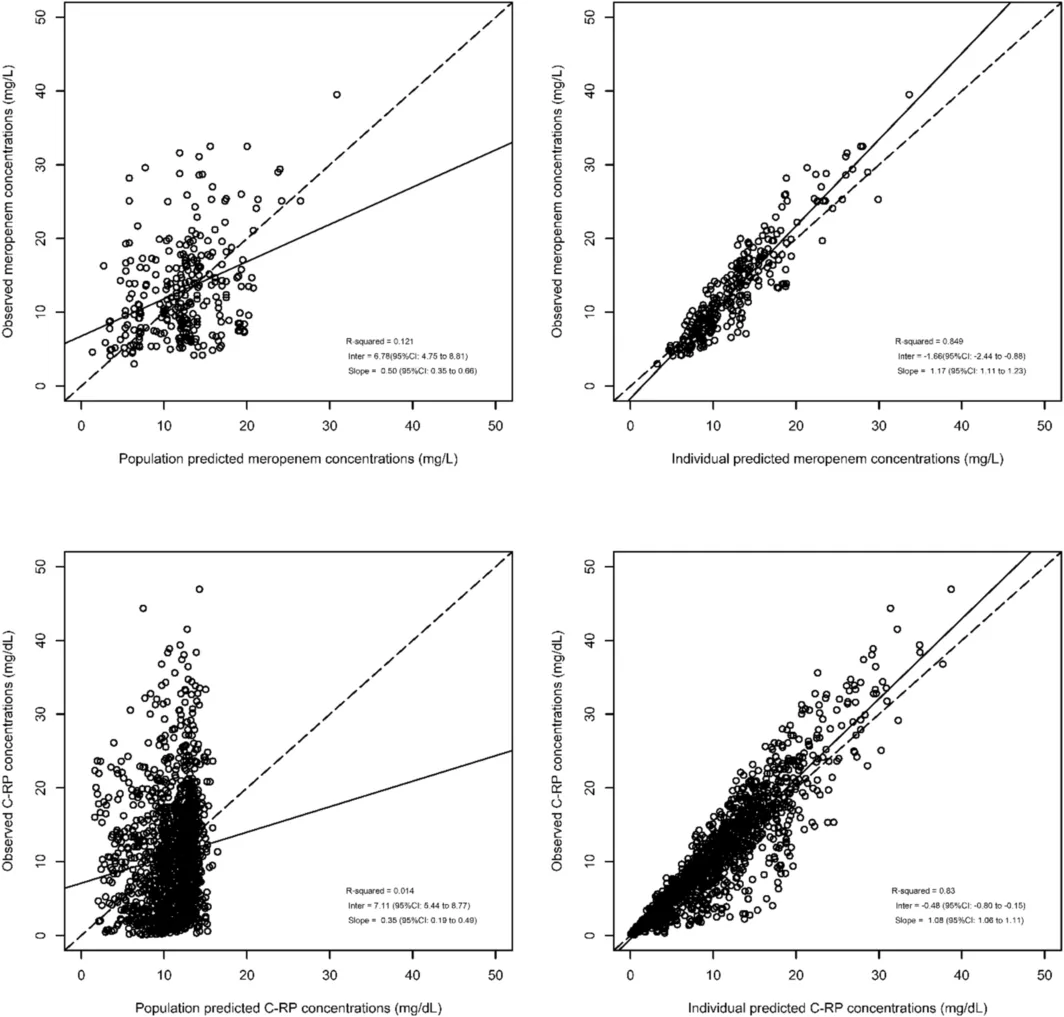
Goodness‐of‐fit plots for the population pharmacokinetic and pharmacodynamic model. Shown are meropenem observed versus population‐ and versus individual‐predicted concentrations (upper panels), and C‐RP observed versus population‐ and versus individual‐predicted concentrations (bottom panels). The solid black line is the line of reference. The dashed line is the line of regression between observed and predicted concentrations.

**FIGURE 3:**
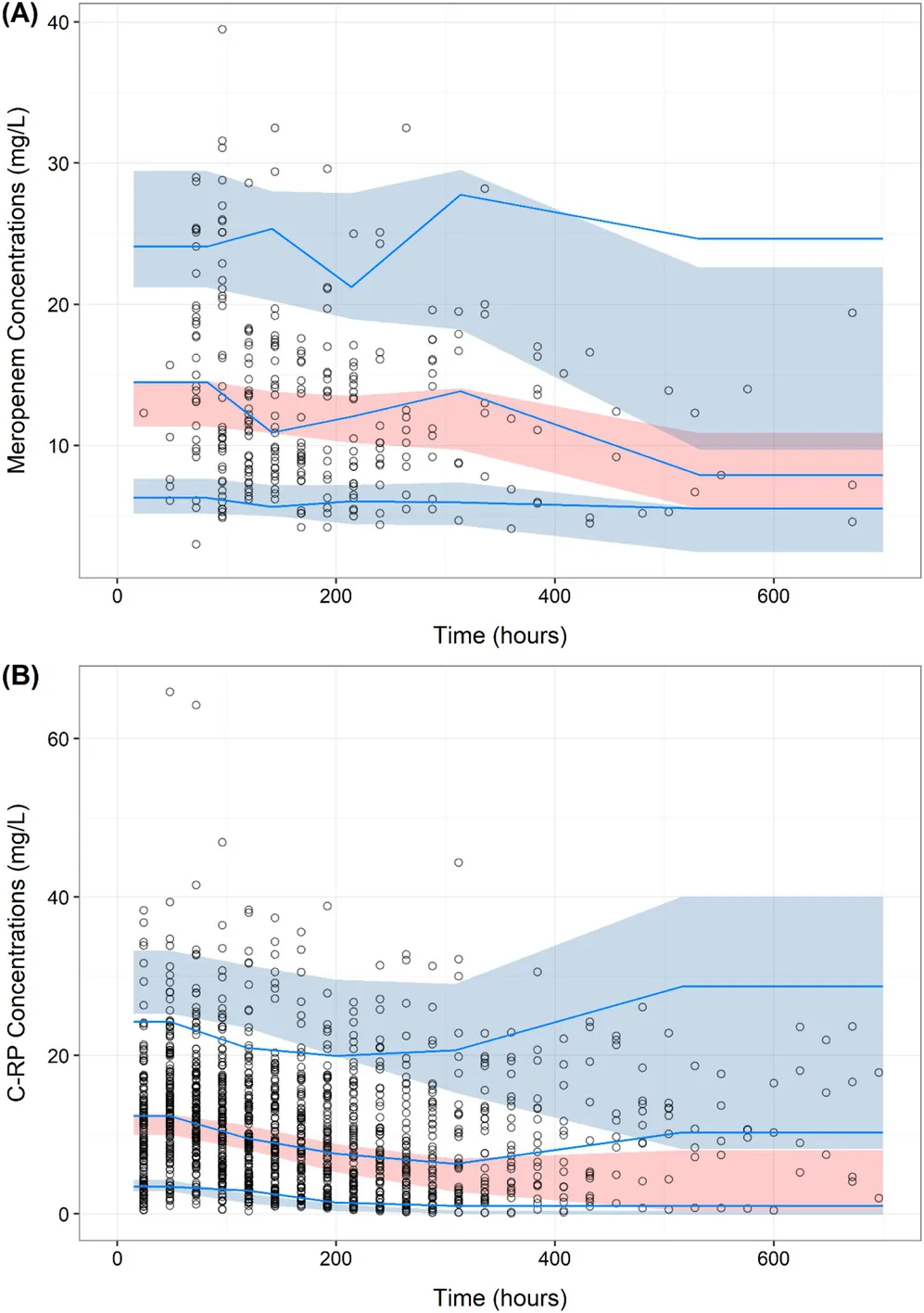
Prediction‐corrected visual predictive check for the population (A) pharmacokinetic and (B) pharmacodynamic models. Three prediction intervals, estimated across 10 000 simulated data, are displayed as coloured areas (pink for the 50th percentile and blue for the 10th and 90th percentiles). Each prediction interval is computed with a level of 90%. The continuous blue lines indicate the 10th, 50th and 90th percentiles for observed data.

The median population pharmacokinetic estimate of meropenem CL was 13.34 L/h with an interindividual variability of 45%. The parameter estimates of the PK/PD model are summarized in Table 2. Model parameters were estimated with good precision as RSE% was less than 30% for most of them.

**TABLE 2:** Summary of the population pharmacokinetic and pharmacodynamic model.

	PK/PD model		Bootstrap results	
Parameter	Value	%*RSE*	Median	5th–95th percentiles
Fixed effects				
*V* (L)	28.5	—	—	—
*CL* (L/h)	4.99	15.4	4.86	3.95–7.23
*β*_eGFR	0.009	17.7	0.0093	0.0054–0.0115
*R*0 (mg/dL)	5.82	13.2	7.08	3.85–9.28
*k*_out_ (h^−1^)	0.02	4.86	0.02	0.02–0.02
*IC*_50_ (mg/L)	41.5	23.6	79	23.72–405.58
*k*_in_ (mg/dL*h^−1^)	0.49	6.09	0.42	0.30–0.56
*k* (h^−1^)	0.0037	15.7	0.0035	0.0019–0.0050
Random effects				
*ω* for *V*	1.64	12.7	1.61	1.27–1.92
*ω* for CL	0.45	7.34	0.45	0.38–0.49
*ω* for R0	1.25	8.35	1.14	0.92–1.39
*ω* for *k*_out_	0.21	22.4	0.20	0.13–0.36
*ω* for *IC*_50_	1.68	11.2	2.05	1.42–3.13
*ω* for *k*_in_	0.47	10.2	0.48	0.36–0.65
*ω* for *k*	1.51	7.59	1.56	1.26–2.08
Residual variability				
*b*1	0.27	5.99	0.26	0.24–0.29
*a*2	0.11	24.6	0.10	0.02–0.22
*b*2	0.31	2.88	0.31	0.28–0.34

Abbreviations: %*RSE*, relative standard error of the population estimates; *a*2, constant residual error for PD model; *b*1, proportional residual error for PK model; *b*2, proportional residual error for PD model; CL, clearance; *IC*_50_, half maximal inhibitory concentration of meropenem; *k*, constant for upward or downward trend of C‐RP; *k*_in_, generation rate constant of C‐RP; *k*_out_, degradation rate constant of C‐RP; *R*0, baseline C‐RP level; *V*, volume of distribution.

Individual fitting of C‐RP concentrations of the population (*n* = 141 patients) allowed to identify 8.5% (12/141) fast C‐RP response patients, 15.6% (22/141) slow C‐RP response patients, 74.5% (105/141) no C‐RP response patients and 1.4% (2/141) biphasic response patients. Some typical individual PD fittings of each scenario are reported in Figure S7. Among patients with available clinical outcome, 1.4% (7/70) were fast C‐RP response patients, 17.1% (12/70) were slow C‐RP response patients, 70% (49/70) were no C‐RP response patients, and 2.9% (2/70) were biphasic response patients.

Univariate and multivariate linear regression analysis of factors associated with favourable clinical outcome is reported in Table 3. At multivariate analysis, the only variable positively associated with favourable clinical outcome was C‐RP fast and slow response pattern (*p* = .006). The proportions of patients having positive or negative clinical outcome across different factors are summarized in Table S1. Favourable clinical outcome was observed in 100% (19/19) of patients with fast and slow response pattern and in 64.7% (33/51) of patients with non‐response or biphasic pattern.

**TABLE 3:** Univariate and multivariate analysis of variables associated with favourable clinical outcomes in patients with febrile neutropenia (*n* = 70).

Variable	Univariate analysis		Multivariate analysis	
	Unstandardized *β* coefficient (95% CI)	*p*	Unstandardized *β* coefficient (95% CI)	*p*
Age (years)	.003 (−0.004, 0.010)	.373		
Gender	−.027 (−0.247, 0.194)	.810		
BMI (kg/m^2^)	.023 (−0.006, 0.052)	.115	.013 (−0.016, 0.042)	.380
eGFR (mL/min/1.73 m^2^)	.001 (−0.004, 0.003)	.921		
Acute leukaemias	−.064 (−0.301, 0.173)	.592		
Treatment duration (days)	.005 (−0.013, 0.022)	.607		
Treatment with antifungal agents	−.265 (−0.896, 0.367)	.406		
Treatment with anti‐Gram‐positive agents	−.213 (−0.455, −0.007)	.043	−.148 (−0.376, 0.080)	.199
C‐RP value at baseline	.001 (−0.007, 0.009)	.866		
C‐RP value at peak	−.005 (−0.014, 0.004)	.258		
C‐RP fast and slow response pattern	.353 (0.131, 0.575)	.002	.318 (0.095, 0.541)	.006

*Note*: C‐RP fast and slow response pattern correspond to C‐RP reduction at Day 4 < 0.4 and between 0.4 and 0.8 from baseline, respectively.

### 3.3 Monte Carlo simulation

Figure 4 shows the reduction or increase from baseline of the median C‐RP profile in relation to the three different patterns of C‐RP, namely, (i) fast response patients, (ii) slow response patients and (iii) non‐response or biphasic patients. Simulations showed that median C‐RP reduction at Day 4 for patients with fast and slow response patterns was 84.9% and 67.6%, respectively, whereas reduction greater than 90% from baseline was achieved by Days 6 and 10, respectively. On the contrary, non‐response and biphasic patients had a median C‐RP peaking at Day 3, followed by a gradual C‐RP reduction which reached baseline value at Day 13. Median C‐RP at Day 4 was increased by 155% with respect to baseline value.

**FIGURE 4:**
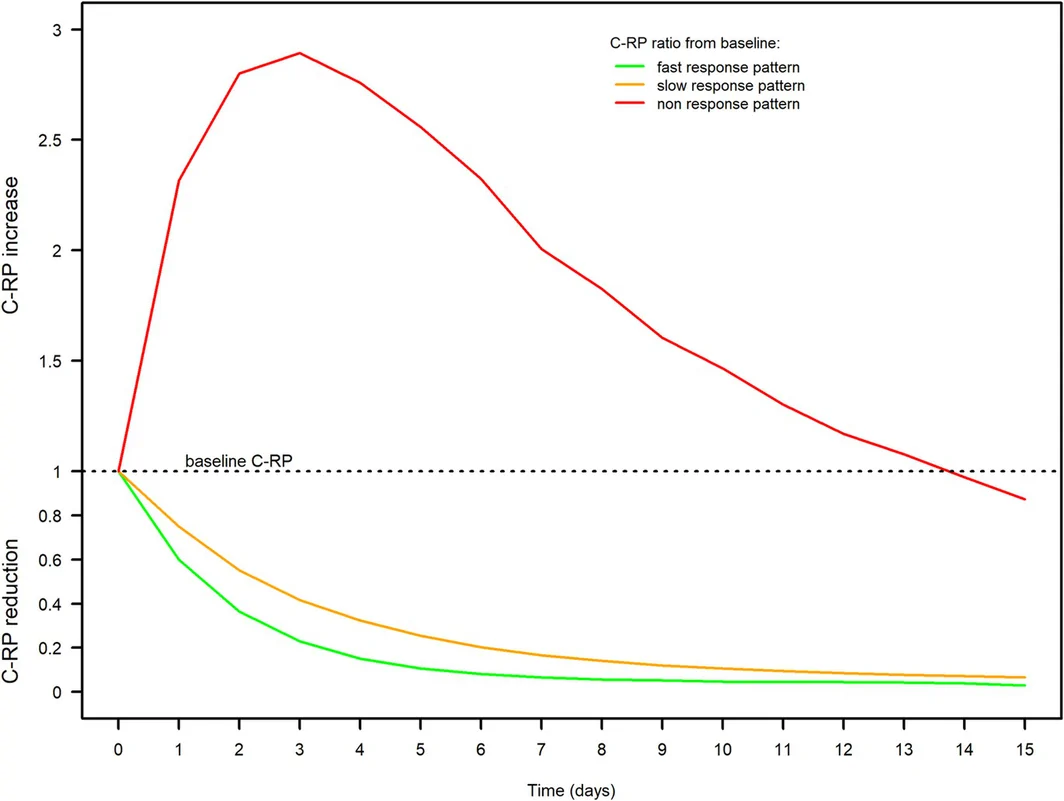
Reduction or increase of estimated median C‐RP over time after administering meropenem dosages that ensured a Css/MIC > 4 at the clinical breakpoint of *Pseudomonas aeruginosa* in relation to the different patterns of C‐RP ratio from baseline at Day 4, namely, fast response pattern (C‐RP ratio declining to <0.4 by Day 4), slow response pattern (C‐RP ratio declining to >0.4 but <0.8 by Day 4), and non‐response pattern (C‐RP persistently >0.8 at Day 4 and/or increasing).

A decision tree supporting clinicians in understanding how C‐RP ratio behaviour at Day 4 may impact early treatment response to meropenem is depicted in Figure 5.

**FIGURE 5:**
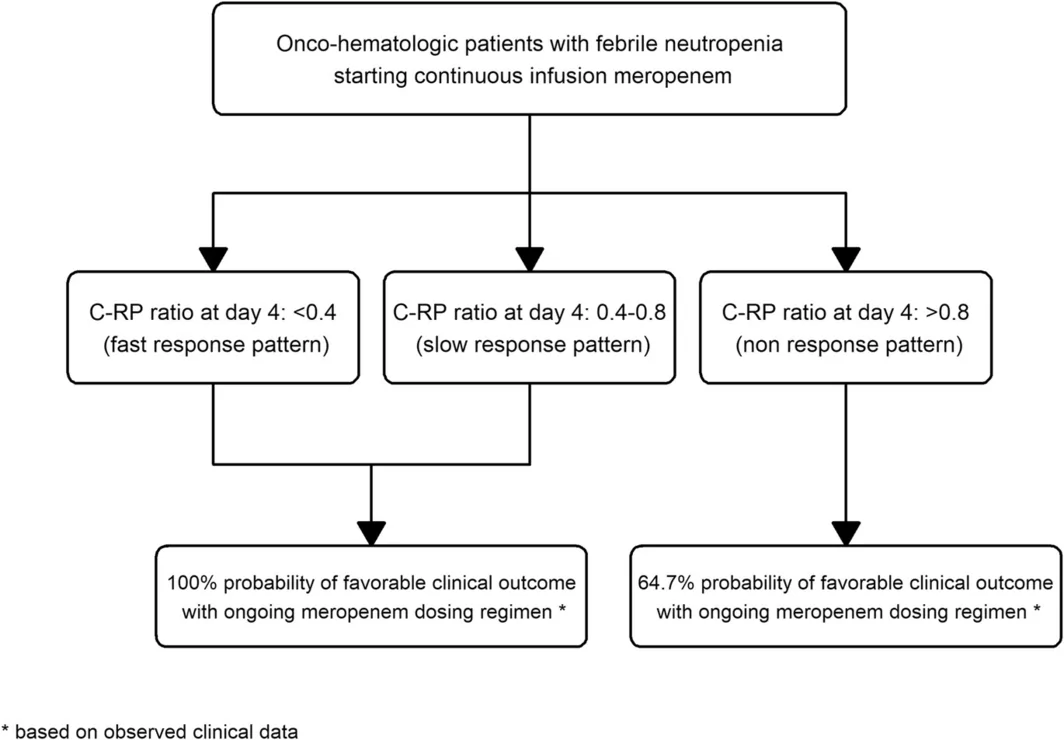
Decision tree supporting clinicians in understanding how C‐RP ratio behaviour at Day 4 may impact early treatment response to meropenem, based on observed clinical data.

## 4 DISCUSSION

In this study, a population PK/PD modelling based on real‐life clinical data was developed to characterize the relationship between meropenem exposure and C‐RP trend over time in a cohort of onco‐haematologic patients with febrile neutropenia.

The population pharmacokinetic estimates of meropenem in our population were consistent with previously reported data in onco‐haematologic patients. Specifically, our CL estimate of 13.87 L/h was similar to the value of 12.3 L/h reported by Contejean et al.^18^ among 88 onco‐haematologic patients with a median eGFR of mL/min/1.73 m^2^ and to the value of 9.7 L/h reported by Lee et al.^20^ among 57 Korean onco‐haematological patients with a median eGFR of 121 mL/min. Our estimate was also similar to the value of 13.04 L/h reported by a previous work of ours^8^ conducted among 61 febrile neutropenic patients with a median eGFR of 107.3 mL/min/1.73 m^2^ who were treated with 24‐h CI meropenem doses of 2–4 g daily. The PK/PD model adequately fitted both meropenem and C‐RP concentrations. With respect to C‐RP, it is worth noting that both a downward and an upward trend was observed in the population, depending on the patient's response to therapy. To capture this opposite variation of C‐RP, a parameter (*k*) was introduced to characterize the time‐varying generation rate of C‐RP and this significantly improved individual fitting.

In the clinical routine, C‐RP is among the most frequently used parameters that clinicians use to evaluate patient response to bacterial infections. C‐RP may be used for identifying patients who develop sepsis after community‐acquired infections as early as Day 3.^21^ Even if procalcitonin may be considered a better biomarker for the diagnosis of bloodstream infections in critically ill patients,^22^ in those with febrile neutropenia, it is not superior to C‐RP or interleukin‐6.^23^ The study of the relationship between C‐RP and drug exposure by PK/PD modelling has been performed by few authors. Ramos‐Martin et al. developed an inhibitory sigmoid *Emax* model for teicoplanin in neonates linking the parameter AUC/EC_50_ to C‐RP and showed that patients achieving an AUC/EC_50_ ratio of >68.3 had a more consistently lower level of C‐RP.^24^ A similar approach was used by Rawson et al. who showed a significant association between vancomycin AUC/EC_50_ > 19 and prediction of C‐RP at 96–120 h among adult non‐critically ill patients.^25^ Recently, Cojutti et al. applied an inhibitory indirect PK/PD model to describe the relationship between dalbavancin and C‐RP in patients with staphylococcal osteoarticular infection.^26^

However, modelling C‐RP in onco‐haematological patients presents some specific issues that are not commonly observed in other populations. The first is related to the generally low baseline value of C‐RP.^11^ The second is related to the impaired immune system that may delay the clearance of infections. Both these aspects may explain the slow decrease of C‐RP over time that we observed. Third, C‐RP may be affected by the underlying onco‐haematological disease, with non‐Hodgkin lymphomas being generally characterized by higher C‐RP levels than acute leukaemias,^27^ although this specific aspect did not emerge in our population PK/PD model. All these criticalities may account for the fact that patients who experience C‐RP reduction from baseline in the first week of treatment are a minority. However, a reduction of C‐RP is associated with positive clinical outcome in our population. Thus, our findings suggest that C‐RP may be used to monitor treatment outcome and that at least 4 days are necessary for assessing response to meropenem treatment based on C‐RP. Combining C‐RP with other biomarkers of response such as body temperature or interleukin‐6 may increase the prediction accuracy of early response to antibiotic therapy.

We acknowledge different limitations of our study. First, its retrospective nature did not allow us to collect a homogeneous number of samples for pharmacokinetic and pharmacodynamic analysis, and this might have contributed to the high variability in some population parameters. Second, we recognize that C‐RP may be biased by some pathophysiological conditions such as the severity of the underlying onco‐haematological disease and the state of the immune system. Third, the absence of a control group precluded us from computing the proportion of the meropenem treatment effect on the clinical outcome explained by changes in C‐RP. Finally, we cannot rule out that other factors other than the C‐RP trend may have contributed to the observed clinical response.

In conclusion, our model is the first that adequately described the inhibitory effect of meropenem on C‐RP production in onco‐haematological patients with febrile neutropenia. Our results showed that C‐RP dynamics is associated with treatment outcome. Specifically, when using meropenem dosing that ensures optimal PK/PD target attainment for empirical treatment of febrile neutropenia, C‐RP reduction may be either reduced by half after 3–4 days of treatment or increased soon after treatment initiation with a median peak at Day 3. In order to detect an early response to therapy and possibly to establish its role in patient clinical outcome, C‐RP should be associated with other biological parameters. This study may represent the first step for further clinical investigations on the use of biomarkers in a population highly susceptible to developing infective complications that are oftentimes the first cause of mortality.

## AUTHOR CONTRIBUTIONS

Chun Liu collected the data, performed the data analysis, and wrote the initial draft of the manuscript. Pier Giorgio Cojutti conceptualized the study, performed the data analysis, and wrote the initial and final versions of the manuscript. Coen van Hasselt conceptualized the study, performed the data analysis, and supervised the study. Michele Bartoletti, Maddalena Giannella, Pier Luigi Zinzani, and Pierluigi Viale conceptualized and supervised the study. Federico Pea conceptualized the study, supervised the study, and wrote the final version of the manuscript.

## CONFLICT OF INTEREST STATEMENT

P.G.C. received fees from Angelini, Shionogi, Pfizer and MSD outside of the submitted work. F.P. participated in the speakers' bureaus of Angelini, BeiGene, Gilead, Menarini, MSD, Pfizer, Sanofi‐Aventis and Shionogi outside the submitted work and acted as a consultant for Advanza Pharma, Angelini, Gilead, MSD, Pfizer and Shionogi outside the submitted work. P.V. acted as a consultant for bioMérieux, Gilead, Merck Sharp and Dohme, Nabriva, Nordic Pharma, Pfizer, Thermo‐Fisher and Venatorx and received payment for serving on the speakers' bureaus for Correvio, Gilead, Merck Sharp and Dohme, Nordic Pharma and Pfizer outside the submitted work. The other authors report no potential conflicts of interest for this work.

## Supporting information


**Table S1** Univariate logistic regression analysis comparing patients with positive versus negative clinical outcome (*n* = 70).
**Figure S1**. Scatter plot of the individual weighted residuals (IWRES) versus time (left panel) and IWRS versus individual predictions (right panel) for the pharmacokinetic component.
**Figure S2**. Scatter plot of the individual weighted residuals (IWRES) versus time (left panel) and IWRS versus individual predictions (right panel) for the pharmacodynamic component.
**Figure S3**. Distribution of the individual weighted residuals (IWRES) and of the normalized prediction distribution error (NPDE) for the pharmacokinetic component.
**Figure S4**. Distribution of the individual weighted residuals (IWRES) and of the normalized prediction distribution error (NPDE) for the pharmacodynamic component.
**Figure S5**. Numerical predictive check (NPC) for the pharmacokinetic component.
**Figure S6**. Numerical predictive check (NPC) for the pharmacodynamic component.
**Figure S7.** Individual fitting of the pharmacodynamic model with full inhibition of C‐RP production for representative cases of fast response, slow response, no response and biphasic response. The blue dots represent C‐RP concentrations. The solid line is the line of fitting.

## Data Availability

The datasets used and/or analysed during the current study are available from the corresponding author on reasonable request.
