# Supplementary material for: Relationship between continuous infusion meropenem PK/PD target attainment and C‐reactive protein dynamics in onco‐haematologic patients with febrile neutropenia
Source: Br J Clin Pharmacol. 2026 Jul 2;92(10):3646–55. doi: 10.1002/bcp.70667 (PMC13619083; doi:10.1002/bcp.70667)
Supplement: Supplementary file 1 [file BCP-92-3646-s001.docx]

**Supplemetal material**

Relationship between continuous infusion meropenem PK/PD target attainment and C-reactive protein dynamics in onco-hematologic patients with febrile neutropenia

Chun Liu, Pier Giorgio Cojutti, Coen van Hasselt, Michele Bartoletti, Maddalena Giannella, Pierluigi Zinzani, Pierluigi Viale, Federico Pea

| **Table S1.** Univariate logistic regression analysis comparing patients with positive vs. negative clinical outcome (n=70) | | | |
| --- | --- | --- | --- |
| Variables | Positive  clinical outcome (n=52) | Negative  clinical outcome (n=18) | Univariate  p-value |
| Age >65 yrs | 16 (30.8) | 4 (22.2) | 0.491 |
| Gender (male) | 33 (63.5) | 12 (66.7) | 0.807 |
| BMI>25 (kg/m^2^) | 22 (42.3) | 5 (27.8) | 0.279 |
| eGFR<60 (mL/min/1.73m^2^) | 8 (15.4) | 4 (22.2) | 0.382 |
| Acute Leukemia | 37 (71.2) | 14 (77.8) | 0.587 |
| Treatment with antifungal agents | 50 (96.2) | 18 (100) | 1.000 |
| Treatment with anti Gram-positive agents | 33 (63.5) | 16 (88.9) | 0.057 |
| C-RP fast and slow response pattern | 19 (36.5) | 0 (0) | **0.002** |
| BMI, body mass index; eGFR, estimated glomerular filtration rate. | | | |

**Figure S1**. Scatter plot of the individual weighted residuals (IWRES) vs. time (left panel) and IWRS vs. individual predictions (right panel) for the pharmacokinetic component

**Figure S2**. Scatter plot of the individual weighted residuals (IWRES) vs. time (left panel) and IWRS vs. individual predictions (right panel) for the pharmacodynamic component

**Figure S3**. Distribution of the individual weighted residuals (IWRES) and of the normalized prediction distribution error (NPDE) for the pharmacokinetic component

**Figure S4**. Distribution of the individual weighted residuals (IWRES) and of the normalized prediction distribution error (NPDE) for the pharmacodynamic component

**Figure S5**. Numerical predictive check (NPC) for the pharmacokinetic component

**Figure S6**. Numerical predictive check (NPC) for the pharmacodynamic component

**Figure S7.** Individual fitting of the pharmacodynamic model with full inhibition of C-RP production. for representative cases of fast response, slow response, no response and biphasic response. The blue dots represent C-RP concentrations. The solid line is the line of fitting.

Fast response

Slow response

No response

Biphasic response
